# Availability of medications for opioid use disorder in outpatient and inpatient pharmacies in South Florida: a secret shopper survey

**DOI:** 10.1186/s13722-022-00346-x

**Published:** 2022-11-18

**Authors:** Alina Syros, Maria G. Rodriguez, Andrew C. Rennick, Grace A. Dima, Alexander R. Gibstein, Lauren de la Parte, Matthew G. Hermenau, Katrina J. Ciraldo, Teresa A. Chueng, Hansel E. Tookes, Tyler S. Bartholomew, David P. Serota

**Affiliations:** 1 Division of Infectious Diseases, Department of Medicine, University of Miami Miller School of Medicine, 1120 NW 14th St, Suite 851, Miami, FL 33136 USA; 2 Jackson Memorial Hospital, Miami, FL USA; 3 Department of Obstetrics, Gynecology, and Reproductive Sciences, University of Miami Miller School of Medicine, Miami, FL USA; 4 Department of Family and Community Medicine, University of Miami Miller School of Medicine, Miami, FL USA; 5 Department of Public Health Sciences, University of Miami Miller School of Medicine, Miami, FL USA

**Keywords:** Opioid use disorder, Buprenorphine pharmacy availability, Pharmacists, Florida, Buprenorphine providers

## Abstract

**Background:**

Despite the proven efficacy of medications for opioid use disorder (MOUD) and recent reduction in barriers to prescribers, numerous obstacles exist for patients seeking MOUD. Prior studies have used telephone surveys to investigate pharmacy-related barriers to MOUD. We applied this methodology to evaluate inpatient and outpatient pharmacy barriers to MOUD in South Florida.

**Methods:**

Randomly selected pharmacies in South Florida (Miami-Dade, Broward, and Palm Beach Counties) were called using a standardized script with a “secret shopper” approach until 200 successful surveys had been completed. The primary outcome was the availability of any buprenorphine products. Second, a list of all 48 acute care hospitals within the aforementioned counties was compiled, and hospitals were contacted by telephone using a second structured script.

**Results:**

A total of 1374 outpatient pharmacies and 48 inpatient pharmacies were identified. 378 randomly selected outpatient pharmacies were contacted to accrue 200 successful calls (53% success rate). All 48 inpatient pharmacies were contacted to successfully complete 25 inpatient surveys (52%). Of the 200 outpatient pharmacies contacted, 38% had any buprenorphine available. There was a significant difference in buprenorphine availability by county, with Miami-Dade having the least availability and Palm Beach having the most availability (27% vs. 47%, respectively; p = 0.04). Of the 38% with buprenorphine available, 82% had a sufficient supply for a two-week prescription of buprenorphine 8 mg twice daily. Of the pharmacies that did not have buprenorphine, 55% would be willing to order with a median estimated time to receive an order of 2 days (IQR 1.25–3 days). Of the 25 surveyed inpatient pharmacies, 88% reported having buprenorphine on inpatient formulary, and 55% of hospitals had at least one restriction on ordering of buprenorphine beyond federal regulations.

**Conclusions:**

The results of this study highlight significant pharmacy-related barriers to comprehensive OUD treatment across the healthcare system including both acute care hospital pharmacies and outpatient community pharmacies. Despite efforts to increase the number of MOUD providers, there still remain downstream obstacles to MOUD access.

## Background

The United States (U.S.) continues to experience an alarming and increasing rate of annual drug overdose deaths, with 108,000 in 2021 [1]. In Florida, 68% of all drug overdoses in 2020 involved opioids, and overdoses involving highly potent synthetic opioids, such as fentanyl, continue to drive the crisis [2]. Beyond increasing opioid-related overdose deaths, injection drug use-associated (IDU) infectious diseases, including bacterial, viral, and fungal infections, are simultaneously escalating and contributing to significant morbidity, mortality, and healthcare expenditures [3–5]. In 2017, South Florida (Miami-Dade, Broward, and Palm Beach counties) experienced over 4,000 acute care hospitalizations attributable to injection-related infections with an estimated cost of more than $100 million [6]. One evidence-based intervention for helping to reduce the intersecting overdose and infectious disease risks associated with IDU are medications for opioid use disorder (MOUD), which include buprenorphine, methadone, and extended-release naltrexone. Compared to non-medication treatments for opioid use disorder (OUD), MOUD is associated with higher treatment retention rates, reduced illicit opioid use, and reduced overdose risk and mortality [7–9].

Although the benefits of MOUD are well-established, access to MOUD remains out of reach to many who stand to benefit. Among American adults with OUD, only 1 in 4 report last-year use of MOUD [10]. Care cascade models evaluating the continuum of OUD treatment for persons with OUD [11]—and persons with opioid injection-associated infectious diseases [12] more specifically—demonstrate numerous gaps in care for treatment-seeking patients. Patient-centered attempts to conceptualize healthcare access generally may be applied to the accessibility of MOUD [13]. Access to MOUD involves a multifactorial process for both patients and providers, including knowledge of MOUD options, ability to visit a provider who can prescribe MOUD, proximity to a pharmacy stocking supply, and appropriate funding for medication [7, 11]. Pharmacy-level barriers also exist that prevent patients from accessing MOUD, with evidence showing that some pharmacists ration buprenorphine and decline to fill legitimate prescriptions [14, 15]. This restricted access to buprenorphine at the pharmacy level may be secondary to stigma towards persons with OUD, insufficent pharmacist training, or due to fear of oversight by the Drug Enforcement Agency (DEA) [9, 16, 17]. It has been reported that some pharmacies have self-imposed buprenorphine restrictions in order to avoid investigations by the DEA, further complicating patient access to MOUD [18–20]. Outpatient pharmacies are a key component of the care cascade for patients with OUD, so understanding the burden and reasoning for these barriers is critical for MOUD implementation.

Less data is available on how acute care hospital pharmacy MOUD stock and policies impact access to MOUD for hospitalized patients with OUD. Federal regulations—as described in 21 CFR § 1306.07—allow for any US hospital-based physician to administer methadone or buprenorphine for relief of opioid withdrawal when patients are hospitalized for medical reasons aside from opioid detoxification [21]. Despite such regulations, states facing high levels of OUD have poor access to medication in the inpatient setting, with states such as New Mexico reporting lack of buprenorphine-naloxone in nearly 50% of acute-care hospitals [22]. In the setting of the emergency department, commonly seen as an opportunity for initiation of MOUD, physicians may be limited by both time and bed capacity, as well as by personal feelings of lacking the readiness to prescribe buprenorphine [23]. In this study, we sought to investigate and document pharmacy-related barriers to MOUD in our South Florida community. Based on similar studies in different regions, we used a secret shopper methodology to quantify the accessibility of MOUD in South Florida pharmacies—both outpatient and inpatient—with the goal of advocating stakeholders to increase the availability of MOUD across our healthcare landscape [9, 16, 22].

## Methods

### Study design

We aimed to describe access to MOUD across the continuum in South Florida (Miami-Dade, Broward, Palm Beach Counties) with a focus on pharmacy-related barriers. We evaluated access in both outpatient and inpatient pharmacies by conducting telephone-based surveys using standardized scripts to assess the availability, formulation, and quantity of buprenorphine or methadone (inpatient pharmacies only). The survey scripts were developed by the research team and refined after a pilot phase of 10 outpatient and 10 inpatient pharmacies. Due to the lack of collection of any patient or survey respondent's personal information, the study was deemed institutional review board (IRB)-exempt by the University of Miami IRB.

### Telephone outpatient pharmacy survey

A list of all outpatient pharmacies was compiled using the county office database for community pharmacies in Miami-Dade, Broward, and Palm Beach Counties [24]. All 1,374 outpatient pharmacies’ contact information was imported into a Microsoft Excel document where the list of pharmacies was randomized. Randomization was not stratified by county or pharmacy type (independent vs. chain). Attempts were made to contact each pharmacy until 200 surveys were successfully completed. Based on Hill and colleagues estimation of 42% buprenorphine availability in Texas, we estimated that a sample size of 200 would allow us to detect a 20% absolute difference in availability by county or chain/independent status [9]. Additionally, we wanted to use a small enough sample that all calls could be placed in as short a time period as feasible. Telephone calls were placed using a structured script developed for outpatient pharmacies (Appendix A). During the calls, research team members identified themselves as medical students working for a physician planning to prescribe buprenorphine-naloxone for a patient but wanting to ensure availability prior to placing the order. Information was obtained from the pharmacist on duty preferentially or otherwise a pharmacy technician. Telephone calls were completed by six medical students (AS, GR, AG, GD, AR, LD) on weekdays (Monday to Friday) during the business hours of 8AM to 5PM between July and October 2021. Pharmacies were contacted using the telephone numbers listed on each pharmacy’s website. Three attempts were made on different days to contact each pharmacy. Independent variables included county, pharmacy company, and pharmacy type (independent or chain). Pharmacies with five or more locations were categorized as chain pharmacies. Pharmacies with less than five locations were categorized as independent pharmacies [9]. Pharmacies were excluded from the study if they were specialty pharmacies, Veterans Affairs pharmacies, or confirmed to be out of operation. Pharmacies that refused to provide information or were unable to be contacted after three separate attempts on three separate days were considered non-responders. The primary study outcome was the availability of any buprenorphine product for OUD. Secondary outcomes included whether they had 14 days of buprenorphine-naloxone 8–2 mg films or tablets, dosed twice daily (≥ 28 films or tablets total), had alternative formulations such as buprenorphine monoproduct, willingness to order buprenorphine, and projected length of time to receive the medication.

### Telephone inpatient pharmacy survey

A list of all inpatient acute care hospitals was compiled using the American Hospital Directory within Miami-Dade, Broward, and Palm Beach Counties [25]. Hospitals were excluded from the study if they were specialty hospitals, long-term care hospitals, or confirmed to be out of operation, yielding a total of 48 inpatient acute care hospitals meeting inclusion criteria. Attempts were made to contact all 48 inpatient pharmacies. Data were not stratified by county. Telephone calls were placed to each pharmacy using a second structured script developed for the inpatient pharmacies (Appendix B). Pharmacies were contacted using the telephone numbers listed on each hospital’s website, by calling the hospital operator and requesting a transfer to the inpatient pharmacy, or through personal contacts at local hospitals. Three attempts were made on different days to contact each pharmacy. For pharmacies where information could not be obtained, an email was sent distributing a survey including the same questions from the script. Pharmacies that refused to provide information or were unable to be contacted after three separate attempts on three separate days were considered non-responders. We sought to describe the availability of any buprenorphine product on inpatient formulary, availability of methadone, and any restrictions on inpatient prescribing. These restrictions included provider type, provider specialty, specific clinical indications, and requirement of X-waiver.

### Statistical analyses

Categorical variables were presented as the number and percentage overall and in each group and were compared between pharmacy type (chain vs. independent) and between counties using the chi-squared test for independence. For comparison of access between different chains, only chains with 10 or more surveys were included. The estimated time to delivery of buprenorphine, if it were to be ordered, was presented as the median and interquartile range (IQR). All statistical analyses were performed using SAS 9.4 statistical software (SAS Institute, Cary, NC).

## Results

There were 1374 registered outpatient pharmacies identified in the tri-county area. Miami-Dade County had the most pharmacies at 644 (46.9%), followed by 430 (31.3%) in Broward County and 300 (21.8%) in Palm Beach. Of these, 378 randomly selected pharmacies were contacted to complete the survey until the study team successfully completed 200 interviews (53% overall response rate). There were significant differences in the response rate by county (p < 0.01) with Palm Beach having the highest response rate (63.0%) and Miami-Dade having the lowest (42.8%). Of the 178 unsuccessful contacts, 96 (53.9%) were confirmed out of operation, 6 (3.4%) were specialty pharmacies, and 75 (42.1%) refused to participate or could not be reached. Of the 200 completed interviews, 77 (38.5%) were from Miami-Dade, 65 (32.5%) were from Broward, and 58 (29%) were from Palm Beach. Characteristics of surveyed pharmacies are presented in Table 1.

**Table 1:** Description of outpatient pharmacies and availability of buprenorphine

	Total, N (%)	Buprenorphine	No Buprenorphine	P-value
Overall	200 (100%)	76 (38%)	124 (62%)	
County				0.04
Miami-Dade	77 (38.5%)	21 (27%)	56 (73%)	
Broward	65 (32.5%)	28 (43%)	37 (57%)	
Palm Beach	58 (29%)	27 (47%)	31 (53%)	
Type				0.06
Independent	40 (20%)	10 (25%)	30 (75%)	
Chain	160 (80%)	66 (41%)	94 (59%)	
Chain*				0.33
Walgreens	67 (33.5%)	33 (49%)	34 (51%)	
Publix	35 (17.5%)	12 (34%)	23 (66%)	
CVS	44 (22%)	21 (48%)	23 (52%)	
Other	14 (7%)	0 (0%)	14 (100%)	

^*^Chains included in statistical test represent chains for which at least 10 surveys were completed. There were 14 other chain pharmacies with fewer than 10 surveys each which were not included in this statistical analysis

**Fig. 1:**
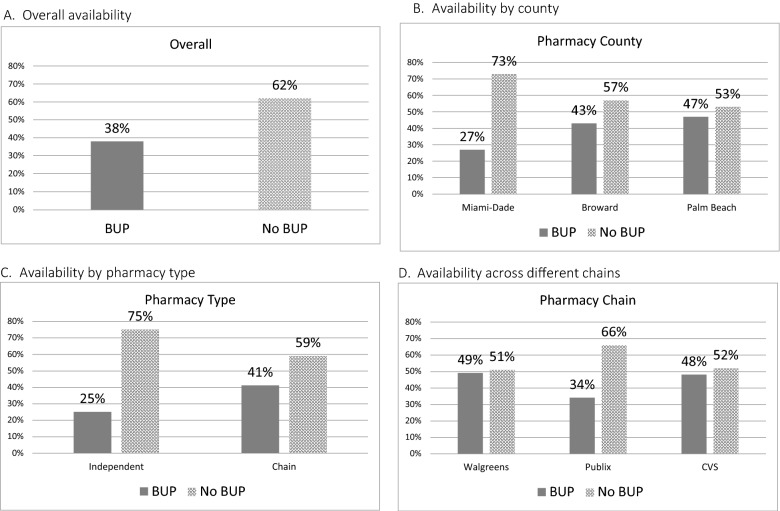
Availability of buprenorphine in outpatient pharmacies in South Florida. **A** Overall availability. **B** Availability by county. **C** Availability by pharmacy type. **D** Availability across different chains. The figures depict rate of South Florida outpatient pharmacies responses regarding current availability of any buprenorphine product at the time of the phone call. **A** Overall availability across responding pharmacies in South Florida. **B** Availability by pharmacies compared by county, P = 0.04 for comparison. **C** Availability by pharmacies compared by whether they are a chain or independent pharmacy, P = 0.06 for comparison. **D** Availability compared between different chain pharmacies, P = 0.33. Chains included in figure and statistical test represent chains for which at least 10 surveys were completed. There were 14 other chain pharmacies with fewer than 10 surveys each which were not included in this statistical analysis. *BUP* buprenorphine

In total, 38% of pharmacies reported having any buprenorphine in stock ready to be dispensed while 62% (n = 124) had none (Table 1; Fig. 1). There was a significant difference in availability of buprenorphine by county (p = 0.04) but not by pharmacy type or specific chain (Table 1; Fig. 1). Of the 38% that did have buprenorphine available, 82% (31% of all surveyed pharmacies) had enough available for a two-week supply of buprenorphine-naloxone 8–2 mg sublingual twice daily (28 films or tablets). There were no pharmacies that stocked buprenorphine monoproduct rather than buprenorphine-naloxone. Of the pharmacies that did not have any buprenorphine, 55% (n = 68) would be willing to place an order with a median estimated time to receive an order of 2 days (interquartile range 1.25–3).

Of the total 48 inpatient pharmacies that were contacted, 23 were classified as non-responders, yielding a 52% response rate. Description of the surveyed inpatient pharmacies is presented in Table 2. Twenty-two (88%) of the inpatient pharmacies reported having a buprenorphine product on inpatient formulary (Table 3). Of those pharmacies, 55% had at least one restriction on inpatient ordering beyond federal regulations (Table 3). All but two hospitals had methadone on inpatient formulary (92%). Sixty-one percent of hospitals with methadone availability had at least one restriction on ordering. The most common restriction for both buprenorphine and methadone was restriction to providers of certain specialties.

**Table 2:** Description of inpatient pharmacies surveyed on buprenorphine and methadone availability

Variable	N (%)
Total	25 (100%)
County	
Miami-Dade	12 (48%)
Broward	7 (28%)
Palm Beach	6 (24%)
Beds	
1–249	7 (28%)
250–424	12 (48%)
≥ 425	6 (24%)
Psychiatric unit	
Yes	11 (44%)
No	14 (56%)
Buprenorphine available	22 (88%)
Methadone available	23 (92%)

**Table 3:** Restrictions on inpatient buprenorphine and methadone ordering

Medication	Restriction on inpatient ordering*	N (%)
Buprenorphine (n = 22)	No restriction	10 (45%)
	Restriction based on provider specialty**	6 (27%)
	Only if receiving treatment prior to admission	3 (14%)
	Only physicians with X-waiver***	3 (14%)
	Pain management only	1 (5%)
Methadone (n = 23)	No restriction	9 (39%)
	Restriction based on provider specialty**	10 (43%)
	Only if receiving treatment prior to admission	3 (13%)
	Only physicians with X-waiver***	2 (9%)

^*^Restriction categories are not mutually exclusive, some pharmacies reported multiple restrictions

^**^Varying combination of hospitalists, psychiatry, addiction medicine, pain specialists

^***^X-waivers, Drug Abuse Treatment Act of 2000 waiver

## Discussion

Using a telephone survey of randomly selected pharmacies in South Florida, we found that there was limited access to buprenorphine in both the inpatient and outpatient settings. Nearly two-thirds of surveyed outpatient pharmacies had no buprenorphine products stocked and only half of those pharmacies were willing to order the medication. Buprenorphine and methadone were accessible in most inpatient hospital pharmacies (88% and 92%, respectively), but more than half of hospital pharmacies had restrictions on inpatient prescribing beyond any federal regulations. We previously identified the burden of IDU-associated hospitalizations in Florida in the 2017 fiscal year [26]. Within Miami-Dade, Broward, and Palm Beach Counties, there were an estimated 4,343 acute care admissions, with 73% involving suspected injection opioids [27]. These hospitalizations, most-often for severe injection-related infections, are reachable moments to engage PWID with OUD into treatment, specifically with lifesaving MOUD [28]. Availability of MOUD from inpatient and outpatient pharmacies are key components to the MOUD care cascade and lack of access serves as a significant barrier to combatting the infectious disease and OUD syndemic [12, 29].

Access to buprenorphine is critical for the health of persons with OUD, yet we have demonstrated that buprenorphine is not widely available in both the outpatient and inpatient settings. This trend is not unique to South Florida; studies conducted across the U.S. have demonstrated similar barriers to care. A national telephone survey of outpatient pharmacies in US counties with high overdose mortality rates found that 20% of pharmacies did not have available stock to dispense buprenorphine [16]. Kazerouni and colleagues found that independent pharmacies were less likely to stock buprenorphine when compared to chain pharmacies [16]. Among community pharmacies in Texas, only 42% of pharmacies had buprenorphine available, the majority of which were chain pharmacies (52%) [9]. Wide variation in availability of buprenorphine in these studies may be due to geographical or survey methodology differences, but anywhere between the published 20% and 60% of pharmacies not having buprenorphine would limit access to a large portion of the population in need of these medications. As evidenced by studies evaluating pharmacists’ perceptions of buprenorphine prescribing, stigma may have influenced the findings of this study. This may include a lack of fundamental knowledge regarding MOUD, pharmacists’ concerns regarding the diversion potential of such medications, as well as diminished trust between pharmacists and physicians due to perceived overprescribing of opioids and fear of DEA investigation due to opioid ordering thresholds [15, 30]. Review of the literature regarding this topic has demonstrated a correlation between advanced training in MOUD with increased confidence and improved counseling when treating patients with OUD [31]. Pharmacists serve an instrumental role in the improvement of access to medication, thus making further training for pharmacists in South Florida a potential window of opportunity for improved treatment outcomes. Our data document some, but not all of the barriers that patients encounter at the pharmacy level. Other relevant barriers include access to forms of government identification, the ability to pay for medications out of pocket, and access to transportation to the pharmacy.

We also demonstrated that limitations exist in the inpatient setting. A study conducted in acute care hospitals in New Mexico reported that 46% of hospitals did not have buprenorphine-naloxone as part of their inpatient formularies [10]. While our study found higher inpatient availability of buprenorphine in South Florida (88%), we identified further hospital policy-level barriers to induction, most often based on provider specialty restrictions. Englander and colleagues described the variety of approaches to hospital-based OUD care, which could include generalists or specialists as the team tasked with OUD treatment [32]. They suggest that formal protocols for opioid withdrawal or treatment are necessary, at a minimum, to help prompt appropriate care. We were unable to identify if these protocols were present in surveyed hospitals. Our study is unique in our description of both inpatient and outpatient pharmacy availability of buprenorphine in the same region. A study by Lewer and colleagues conducted in the United Kingdom demonstrated 4 times the odds of overdose mortality in the 1–2 days after hospital discharge compared to time periods without recent healthcare exposure [33]. These data highlight the critical need to mitigate barriers to MOUD in the transition from hospital to post-hospital period [34–36].

This study has several limitations that affect external validity. In both inpatient and outpatient arms of the study, our survey response rate was approximately 50%. In addition, there were significant differences in the response rate by county. Reasons for inability to complete surveys were not well documented but were primarily due to inability to make contact with a pharmacist. Non-responders may have systematic differences that make them more or less likely to carry MOUD than the responders. All information was based on self-report of survey respondents and policies (inpatient pharmacies) and actual stock were not verified. However, due to the secret shopper methodology for outpatient pharmacies, respondents were led to believe that a physician would imminently send a prescription order, so we believe responses reflect an accurate approximation of medication availability. Pharmacy staff estimation of delivery times for ordered medications could not be verified. Finally, the reasons that pharmacies did not maintain stock of buprenorphine—or were unwilling to order it—were not evaluated in this study but exploring these factors will serve as an important next step in determining how to improve the availability of buprenorphine in outpatient pharmacies.

## Conclusion

The results of this study highlight important local pharmacy-related barriers to comprehensive OUD treatment across the healthcare system including both acute care hospital pharmacies and outpatient community pharmacies. Although efforts to increase access to MOUD in the US have focused on increasing the number of providers who can prescribe buprenorphine—most recently by removal of the requirement to complete 8-h of OUD treatment training—we have shown further downstream obstacles to buprenorphine access [37]. Future studies should aim to better understand the rationale for inpatient pharmacy restrictions on MOUD ordering and lack of robust stock of this time-sensitive treatment in outpatient pharmacies. Additionally, future work should investigate how geographic variability in buprenorphine access could drive ongoing racial and ethnic disparities in buprenorphine access and drug overdose deaths [38, 39].

## Data Availability

The datasets generated during and/or analyzed during the current study are not publicly available due to identification of specific pharmacies but are available from the corresponding author on reasonable request.

## Appendix A

### Outpatient pharmacy script

1. Call the pharmacy, push button to get a representative and request to speak with pharmacist
  - Begin the encounter with: “Hi, I am a medical student calling from the University of Miami. The attending that I am working with is planning to send over a BUP-NX script for a patient and I just wanted to make sure that you have it available before we send it over.”
    i. Do you have Buprenorphine/Naloxone (suboxone) sublingual films available? (yes/no)
      1. **If YES,** enough for a 2-week supply of 8–2 mg BID (28 films)?
        - At least 8 mg? (yes/no)
          i. **If NO**, do you have a 1-week supply available? (yes/no)
            1. At least 8 mg? (yes/no)
2. **If NO**, do you have a 2-week supply of a different formulation of buprenorphine 8 mg BID? (for example, Buprenorphine/Naloxone tablets or Buprenorphine tablets) (yes/no)
  2. **If NO,** would you be willing to have them ordered? (yes/no)
    - **If YES,** how long would it take to obtain a supply?
    - **If NO,** is there a partial supply available until the full amount is available? (yes/no)

## Appendix B

### Inpatient pharmacy script

1. Call the hospital directory, push button to speak with operator and be transferred to the inpatient pharmacy.
  - Begin the encounter with: “Hi, I am a medical student calling from the University of Miami. I am performing a study regarding buprenorphine access and I was hoping to ask a few questions about distribution at your institution.”
    i. Do you have any buprenorphine products on inpatient formulary? [can clarify, Suboxone or Subutex as examples] (yes/no)
2. If YES, which do you have:
  1. Buprenorphine (Subutex)
    - Brand or generic?
    - At least 8 mg?
3. Buprenorphine/naloxone (Suboxone)
  1. Brand or generic?
  2. At least 8 mg?
    b. **If NO** to having it on their formulary: could you obtain it within 24 h if a physician felt it was indicated? (yes/no)
    c. **If YES** to having any formulation, then ask about restrictions
  3. For buprenorphine to treat opioid addiction [may clarify to say OUD, or opioid withdrawal] for hospitalized patients, is prescribing for inpatients restricted to any particular type of provider? (yes/no)
    - Who can prescribe? (examples below)
      i. Psychiatry
      ii. Addiction medicine specialists only
      iii. pain/anesthesia management specialists
      iv. Internal medicine/hospitalists
      v. Emergency medicine
      vi. Only doctors with a DATA 2000 waiver
      vii. No restriction for inpatient prescribing
      viii. For pain control use only
    - Do you have methadone available on the inpatient formulary? (yes/no)
4. **If YES,** are there restrictions of who may prescribe methadone treatment of opioid addiction [may clarify to say OUD, or opioid withdrawal] (yes/no)
  1. Who can prescribe? (examples below)
    - Psychiatry
    - Addiction medicine specialists only
    - Pain/anesthesia management specialists
    - Internal medicine hospitalists
    - Emergency medicine
    - Only doctors with X-waiver
    - No restriction for inpatient prescribing
    - For pain control use only

## Abbreviations

SSP: Syringe services program
SUD: Substance Use Disorder
OUD: Opioid use disorder
MOUD: Medication for opioid use disorder
PWID: Persons Who Inject Drugs
IDU: Injection drug use
